# Non-Catalytic Site HIV-1 Integrase Inhibitors Disrupt Core Maturation and Induce a Reverse Transcription Block in Target Cells

**DOI:** 10.1371/journal.pone.0074163

**Published:** 2013-09-09

**Authors:** Mini Balakrishnan, Stephen R. Yant, Luong Tsai, Christopher O’Sullivan, Rujuta A. Bam, Angela Tsai, Anita Niedziela-Majka, Kirsten M. Stray, Roman Sakowicz, Tomas Cihlar

**Affiliations:** Gilead Sciences Inc., Foster City, California, United States of America; Queensland Institute of Medical Research, Australia

## Abstract

HIV-1 integrase (IN) is the target for two classes of antiretrovirals: i) the integrase strand-transfer inhibitors (INSTIs) and ii) the non-catalytic site integrase inhibitors (NCINIs). NCINIs bind at the IN dimer interface and are thought to interfere primarily with viral DNA (vDNA) integration in the target cell by blocking IN-vDNA assembly as well as the IN-LEDGF/p75 interaction. Herein we show that treatment of virus-producing cells, but not of mature virions or target cells, drives NCINI antiviral potency. NCINIs target an essential late-stage event in HIV replication that is insensitive to LEDGF levels in the producer cells. Virus particles produced in the presence of NCINIs displayed normal Gag-Pol processing and endogenous reverse transcriptase activity, but were defective at initiating vDNA synthesis following entry into the target cell. NCINI-resistant virus carrying a T174I mutation in the IN dimer interface was less sensitive to the compound-induced late-stage effects, including the reverse transcription block. Wild-type, but not T174I virus, produced in the presence of NCINIs exhibited striking defects in core morphology and an increased level of IN oligomers that was not observed upon treatment of mature cell-free particles. Collectively, these results reveal that NCINIs act through a novel mechanism that is unrelated to the previously observed inhibition of IN activity or IN-LEDGF interaction, and instead involves the disruption of an IN function during HIV-1 core maturation and assembly.

## Introduction

Advances in antiretroviral drug development have enabled effective long-term control of HIV-1 infection and the prevention of disease progression. HIV integrase (IN) inhibitors comprise the newest class of approved antiviral agents. The primary and well-characterized role of IN is to catalyze the insertion of viral DNA (vDNA) into the genome of infected cells [1]. Integration is accomplished via two catalytic steps following the completion of reverse transcription: i) 3′-processing that involves trimming the 3′-ends of the vDNA and ii) DNA strand transfer, where IN inserts the vDNA into the host chromosome. Through these events, IN remains bound to the vDNA ends as part of a pre-integration complex (PIC) along with several viral and host proteins [2], [3]. The host protein LEDGF/p75 is critical for vDNA integration because it mediates the tethering of the IN-DNA complex to the host chromatin [4], [5]. The C-terminal IN-binding domain (IBD) of LEDGF/p75 engages the IN protein [6], [7], while the N-terminal elements that harbor a PWWP domain and a pair of AT-hook DNA-binding motifs mediate tethering of the PIC to host chromatin [8], [9]. Depleting LEDGF/p75 levels in the target cells reduces HIV-1 integration efficiency [10]–[13]. In addition, somatic knock-out of LEDGF was shown to severely attenuate the replication of laboratory-adapted HIV-1 strains and completely block the growth of clinical isolates [14].

The direct inhibition of IN catalytic function as well as IN-LEDGF/p75 interaction offer attractive targets for small-molecule antiviral intervention. HIV-1 integrase inhibitors currently in the clinic include the approved drugs raltegravir (RAL; MK-0518) [15], [16] and elvitegravir (EVG; GS-9137) [17], [18], and the newer representative dolutegravir (DTG; S/GSK1349572), which is currently in late-stage clinical development [19], [20]. All three integrase inhibitors bind at the enzyme active site and block integration of vDNA into the host chromosome. Inhibitors that share this mode of action are referred to as IN strand transfer inhibitors (INSTIs).

Advancements in IN structure–function information have enabled the exploration of allosteric inhibitors of HIV-1 IN as an alternate approach to inhibiting viral replication. High-throughput screening together with structure-based rational drug design has yielded molecules with submicromolar antiviral activity [21]–[25]. Allosteric inhibition of IN has been approached in two ways: i) disrupting IN multimerization using small molecules or short peptides that bind the IN dimer interface [26]–[29] and ii) disrupting IN interaction with LEDGF/p75 [21], [27], [30]. Inhibitors that act by these mechanisms are referred to as non-catalytic IN inhibitors (NCINIs). Examples of this class include the LEDGINs [21], tBPQAs [31], and ALLINIs [32], [33], all of which bind the LEDGF-binding pocket at the IN dimer interface. Detailed mechanistic studies recently uncovered a dual mode of action for the NCINIs, wherein the compounds were capable of both blocking IN-LEDGF interaction as well as inhibiting the IN enzymatic activity in a LEDGF-independent manner [31], [32]. In the latter case, NCINI binding promotes IN dimer formation in a manner that prevents IN assembly onto the vDNA ends for 3′ processing [31], [32].

We recently described NCINIs GS-A, -B, and -C with potent *in vitro* antiviral activity both in transformed T-cell lines and primary human peripheral blood mononuclear cells (PBMCs) [31]. Compounds in this series displayed good correlation between the IN dimer promotion activity and the antiviral potency in HIV-infected cell cultures. Mutations conferring HIV-1 resistance to these inhibitors map to the IN dimer interface within and near the LEDGF-binding pocket, confirming IN as the target for NCINIs. Importantly, these compounds were found to inhibit vDNA integration in target cells, albeit at high concentrations (10 µM) [31]. In the present study, we used a series of virologic, biochemical, and imaging techniques to assess whether the interference with the vDNA integration is indeed the primary antiviral mode of action for NCINIs. Surprisingly, our results reveal that although the compounds still act through binding to the IN dimer interface, their antiviral effect is primarily derived during the late stage of virus replication via the promotion of IN multimerization and disruption of proper core maturation. These effects render the progeny virus noninfectious due to an inability to initiate vDNA synthesis following entry into the target cells. Our findings not only demonstrate a novel mode of action for the NCINI class, but also support the previously proposed role of IN protein in the process of HIV assembly and maturation [34].

## Results

### NCINIs Act Predominantly during the Late Stage of HIV Replication Cycle

**Figure 1:**
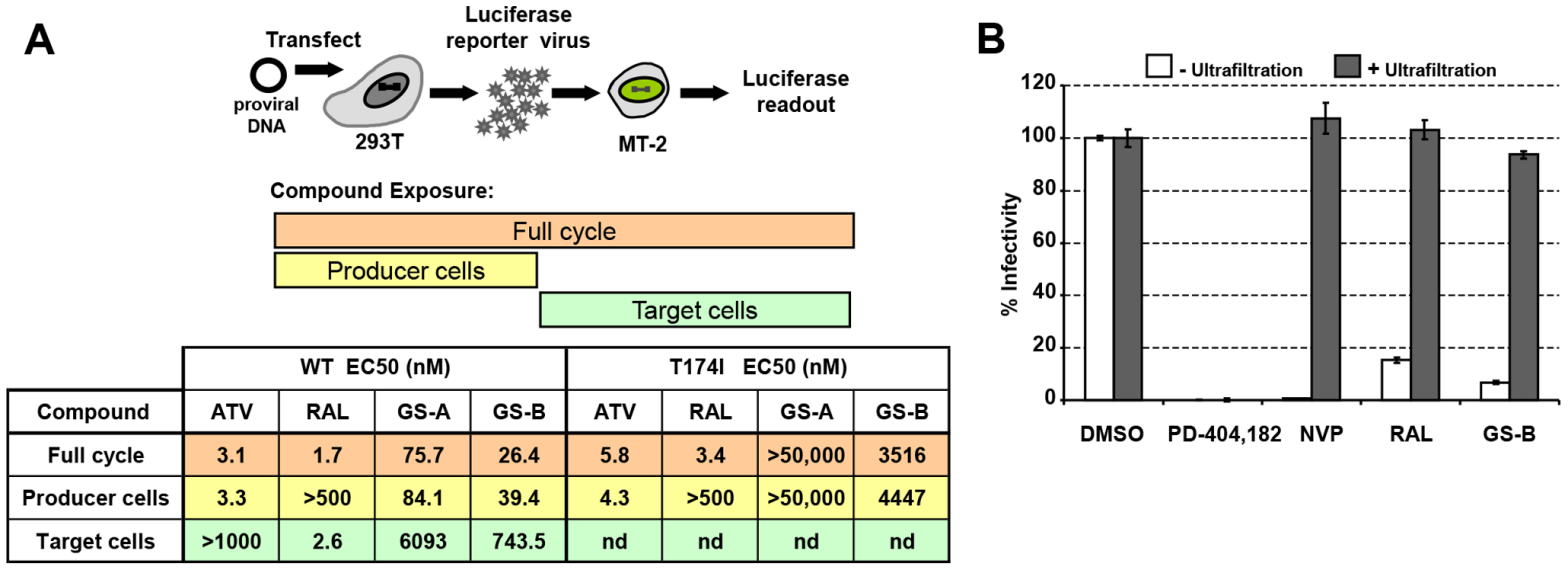
NCINIs act as late-stage HIV-1 inhibitors. (**A**) Schematic overview of the full cycle assay system is shown. Exposure to compounds was limited to the stage of virus production (yellow), target cell infection (green), or both (orange). EC_50_ values for ATV, RAL, GS-A, and GS-B for WT and T174I-IN mutant virus measured under the three compound exposure conditions are tabulated. Results represent a mean of two independent experiments. *nd*, not determined. (**B**) Cell-free virus was incubated with the different classes of inhibitors, and the infectivity was measured before (white bars) and after (gray bars) compound removal via ultrafiltration. Results represent mean ± SD values obtained from triplicate measurements in at least three independent experiments.

To identify stages in the virus life cycle impacted by NCINIs, we utilized a two-part assay system that enabled the measurement of antiviral potency at the late vs early phase of the virus life cycle by altering the presence of compounds in cultures of virus producer and target cells (Figure 1A). As expected, the integration inhibitor RAL was active only when present during infection of the target cells, whereas the late-acting protease inhibitor atazanavir (ATV) exhibited antiviral activity only when present during virus production. Surprisingly, when the NCINIs GS-A and GS-B were restricted to only the target cell infection phase, both compounds showed a marked reduction in potency (80- and 28-fold, respectively) relative to that observed in the full-cycle assay. Importantly, and in striking contrast to RAL, restricting NCINI exposure to the virus-production stage of infection was sufficient to yield activity similar to that observed in the full-cycle assay. In addition, similar late-stage potencies were observed with virus produced from MT-2 T cell lines (data not shown) and closely matched activity observed in multi-cycle assays employing human primary PBMCs [31].

The T174I mutation in IN confers resistance to the NCINI class of inhibitors [31]. The T174I virus is substantially less sensitive to the late-stage inhibition of both GS-A and GS-B, but retains full sensitivity to RAL and ATV (Figure 1A), confirming that the late-stage antiviral effect of NCINIs is still driven by targeting the viral IN protein.

We next investigated whether the late-stage effect of NCINIs involves any virucidal effect on virus progeny. We incubated cell-free mature virus with high concentrations of the inhibitor and then removed unbound compound by repeated dilution and ultrafiltration of the virus sample. While the virucidal agent PD-404,182 [35] caused a permanent loss of infectivity, virus treated with GS-B, RAL, or the reverse transcriptase inhibitor nevirapine retained similar infectivity as the mock-treated virus even after prolonged (up to 26 hours) compound treatment (Figure 1B and data not shown). Collectively, these findings indicate that although NCINIs are capable of blocking infection in target cells via interference with vDNA integration, they act primarily during virus production and/or maturation. Once the virus maturation process is complete, however, the virions become refractory to the action of these inhibitors.

### Late-stage Effect of NCINIs is Unaffected by LEDGF Expression Levels in Virus Producer Cells

**Figure 2:**
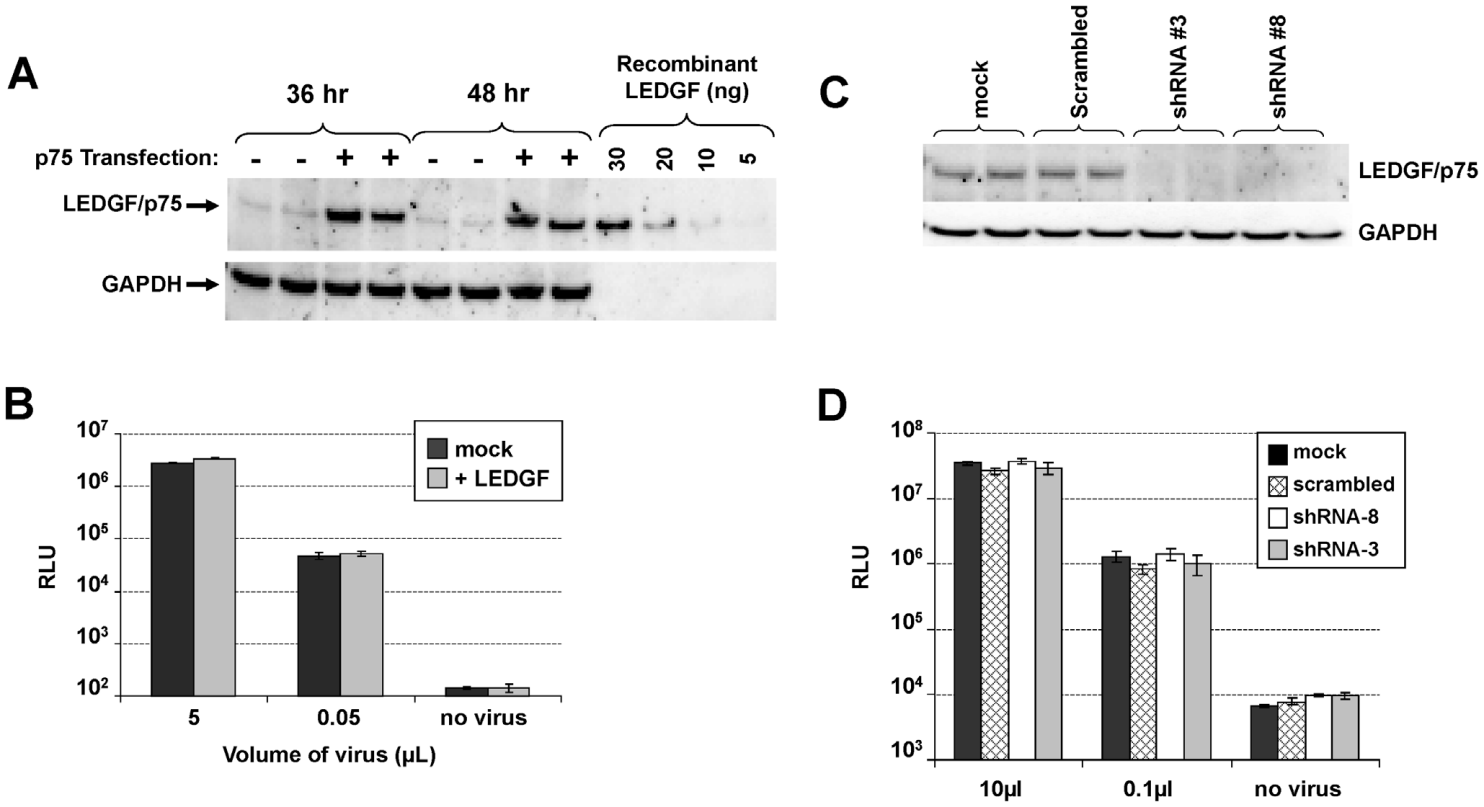
NCINI potency is unaffected by LEDGF expression levels during virus production. (**A**) Western blot analysis of HEK293T cells overexpressing LEDGF during virus production. (**B**) Infectivity of virus produced from mock (black bars) and LEDGF overexpressing (light grey bars) HEK293T producer cells. (**C**) Western-blot analysis of LEDGF expression in HEK293T cells generated from mock transfection or stable transfection with control scrambled or LEDGF-specific shRNAs. (**D**) Infectivity of virus produced from HEK293T cells that were mock transfected (black bars) or stably transfected with scrambled shRNA (hatched bars) or LEDGF shRNA #8 (white bars) and #3 (grey bars). Infectivity measurements for B and D represent mean ± SD values obtained from triplicate measurements from a representative experiment.

While prevailing data suggest that LEDGF binding to IN occurs primarily in target cells, a role of LEDGF-IN interactions in the producer cells has not yet been completely excluded. Given that GS-A and GS-B share the same IN dimer binding pocket as LEDGF, we investigated whether modifying the LEDGF expression levels in the virus producer cells had any effect on NCINI antiviral potency. Transfection of HEK293T cells with the LEDGF expression plasmid yielded sustained, high-level expression of LEDGF, reaching up to 20 times the endogenous levels by 36–48 hours post-transfection (Figure 2A). LEDGF overexpression in the virus producer cells had no discernible impact on virus production or infectivity (Figure 2B).

Virus producer cells overexpressing LEDGF were treated with NCINIs, and the virus was collected only after a high level of LEDGF was reached. Under these conditions, activities of the tested compounds, including both NCINIs, remained unaffected by the LEDGF expression levels in the virus producer cells (Table 1), suggesting a LEDGF-independent late-stage effect of NCINIs.

**Table 1:** LEDGF expression levels during virus production do not influence NCINI late-stage antiviral potency.

Compound	EC50 [nM] (fold change relative to mock control)*					
	LEDGF overexpression		LEDGF knockdown			
	Mock	+ LEDGF transfection	Mock	scrambled RNA	shRNA-3	shRNA-8
RAL	1.01	1.81 (1.8)	3.32	3.84 (1.2)	1.61 (0.5)	1.90 (0.6)
ATV	0.49	0.87 (1.8)	0.69	1.04 (1.5)	1.35 (2)	0.82 (1.2)
GS-A	63.9	58.1 (0.9)	107	81.6 (0.8)	72.5 (0.7)	80.1 (0.7)
GS-B	7.34	11.3 (1.5)	18.4	22.2 (1.2)	14.8 (0.8)	8.62 (0.5)

* Data represent mean of two independent repeats.

To further confirm this observation, we established two independent HEK293T cell lines stably expressing siRNAs targeting different regions of the LEDGF sequence. Both cell lines showed undetectable levels of LEDGF by immunoblot analysis (Figure 2C) and produced normal levels of infectious virus (Figure 2D). Although we cannot rule out the possibility that low levels of LEDGF may still remain in the knock-down cell lines, these data strongly suggest that LEDGF in producer cells is not essential for HIV-1 infectivity, a conclusion consistent with previous reports [12]. The antiviral potency of both NCINIs and the control inhibitors remained unaffected by LEDGF knock-down in virus producer cells (Table 1), further supporting the conclusion that the late-stage inhibitory effect of NCINIs is independent of LEDGF.

### NCINIs Affect Neither the Processing of Gag/Gag-Pol Nor the Virion-associated Reverse Transcriptase Activity

**Figure 3:**
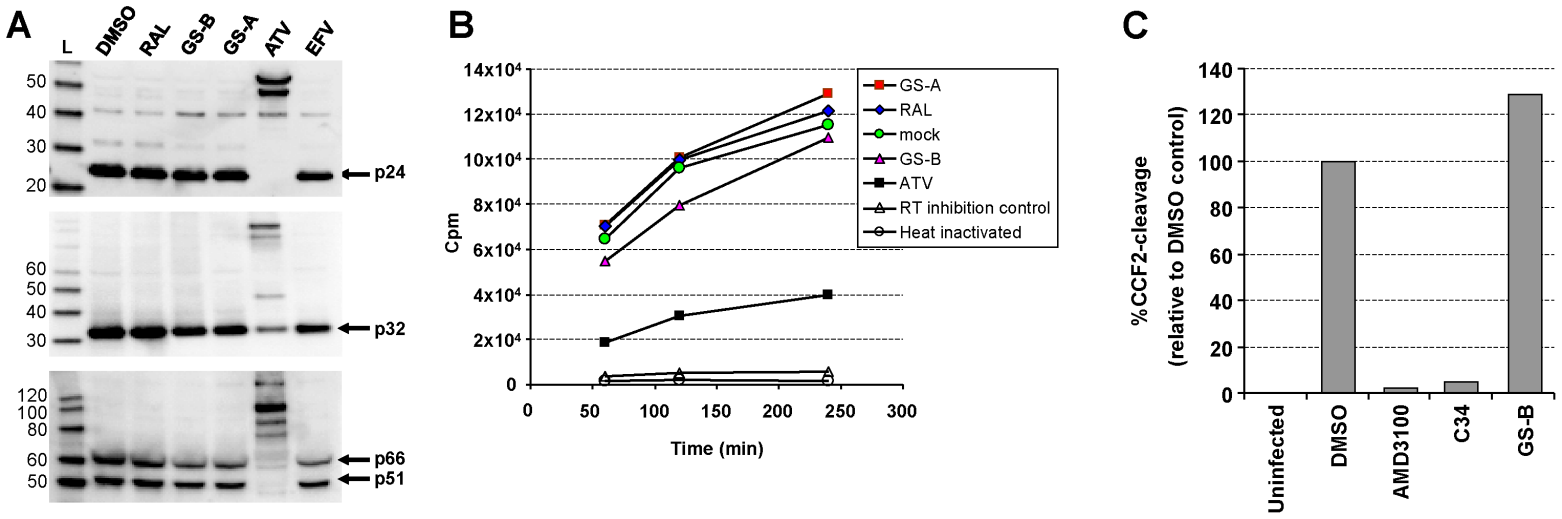
NCINIs do not inhibit Gag-Pol processing, ERT, or target cell entry. (**A**) Western blot analysis of purified HIV-1 particles produced in the presence of different inhibitors at 1 µM concentration. Gag/Gag-Pol processing was assessed using anti-CA (*upper*), anti-IN (*middle*), and anti-RT antibodies (*lower*). (**B**) Equal p24 amounts of virus shown in A were analyzed for virion-associated endogenous reverse transcriptase activity. Values were averaged from duplicate measurements. (**C**) Percentage of cells positive for beta-lactamase activity following infection with Blam-Vpr complemented HIV-1 produced in the presence of DMSO or 1 µM tested compounds. Values are normalized to DMSO-treated control and represent a mean of two independent experiments performed in duplicate.

To assess whether NCINIs alter the packaging or processing of Gag and/or Gag-Pol, progeny virus was generated in the presence of NCINIs and analyzed. Exposure of producer cells to NCINIs did not significantly affect virus yields, as determined by p24 ELISA (data not shown). Furthermore, no differences in the formation of mature p24 capsid (CA), p32 IN and p51/p66 reverse transcriptase (RT) were detected by immunoblot analysis of purified virions produced in the presence or absence of NCINIs (Figure 3A). In contrast, ATV-treated virions showed the expected block in the Gag and Gag-Pol processing.

We additionally analyzed the virus preparations for their virion-associated endogenous RT (ERT) enzymatic activity. The ERT activity relies on the endogenous tRNA primer, viral genomic RNA, and active RT protein packaged within the mature viral particles. As expected, the addition of efavirenz (EFV) or heat inactivation of purified virions fully abolished ERT activity, while a markedly reduced activity was observed in progeny produced in the presence of ATV (Figure 3B). In contrast, no difference in ERT activity was observed between virions produced in the presence of DMSO, RAL, GS-A, or GS-B, indicating that NCINIs did not adversely affect various components of the RT complex.

### NCINIs do not Block Virus Entry

We next tested whether NCINIs affect virus entry into target cells, utilizing the β-lactamase-Vpr virus fusion assay [36]. Virus containing the β-lactamase-Vpr chimeric protein was produced in the presence of GS-B or control compounds and used to infect MT-2 cells. Fusion-mediated virus entry was quantified by monitoring the conversion of β-lactamase substrate reporter dye. Whereas AMD3100 (CXCR4 inhibitor) and C34 peptide (gp41 fusion inhibitor) effectively blocked HIV-1 entry into target cells, no discernible defect in virus entry was observed for virions produced in the presence of NCINIs (Figure 3C). Additionally, the late-stage potency was unaffected by the viral envelope type (HIV-1 gp160 envelope or VSV-G) (data not shown). These combined observations indicate that the block in target-cell infection by NCINI-treated progeny occurs post-entry.

### Progeny Virus Produced in the Presence of NCINIs is Defective in Reverse Transcription

**Figure 4:**
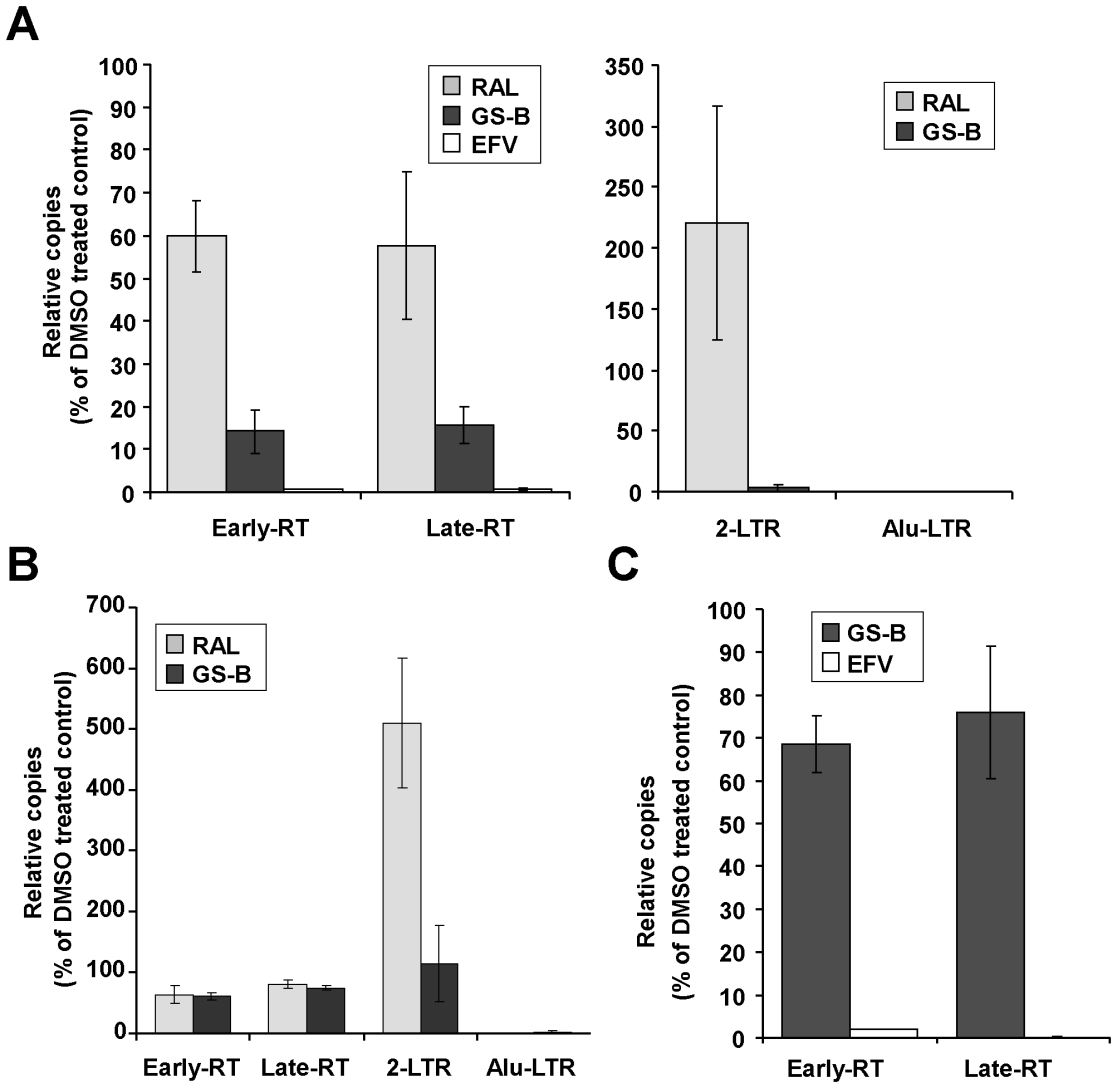
Virus produced in the presence of GS-B is defective in vDNA synthesis in target cells. (**A**) HIV-1 produced in the presence of DMSO, 1 µM RAL (gray bars), 1 µM GS-B (black bars), or 1 µM EFV (white bars) was used to infect MT-2 cells in the presence of the same respective drugs and assayed by quantitative PCR 12 hours post-infection for early and late reverse transcription products (*left panel*), and 24 hours post infection for aborted (2-LTR) and completed (Alu-LTR) integration products (*right panel*). (**B**) Quantitative PCR assessment of reverse transcription and integration products in MT-2 target cells directly infected with HIV-1 in the presence of 1 µM RAL (gray bars) or 5 µM GS-B (black bars). (**C**) Quantitative PCR assessment of early RT and late RT product formation in MT-2 cells infected with T174I virus produced and infected in the presence of GS-B (1 µM) or EFV (1 µM). Results in A-C represent mean ± SD values normalized to DMSO (set to 100%) obtained from quadruplicate infections each assayed in duplicate.

To test whether the late-stage effect of NCINIs still results in a block in vDNA integration, viruses produced in the presence of DMSO, RAL, or GS-B were used to infect MT-2 target cells in the presence of the same compounds. Reverse transcription was monitored by quantifying early and late RT products, and integration by the levels of 2-LTR circles and Alu-LTR products. Treatment of the producer and target cells with RAL (1 µM) resulted in a specific block of integration, as evidenced by elevated levels of abortive 2-LTR circles and undetectable Alu-LTR integration products, but minimal changes to the levels of RT products (Figure 4A). Under the same conditions, the RT inhibitor EFV fully blocked vDNA synthesis. Surprisingly, and in contrast to RAL, treatment of the producer and target cells with 1 µM GS-B caused a profound reduction in the early RT products (Figure 4A). Consistent with previous findings [31], when compound treatment was restricted to target cells alone, GS-B at high concentrations (5 µM) no longer affected reverse transcription but effectively blocked HIV integration (Figure 4B). Treatment of target cells alone with RAL produced a predicted block in integration (Figure 4B). Importantly, the T174I-resistant mutant produced in the presence of NCINIs was refractory to the block in vDNA synthesis (Figure 4C), confirming that the induced RT defect is mediated through binding integrase. Taken together, these data indicate that NCINIs inhibit virus replication by targeting an IN-dependent step during virus production that manifests as a block in vDNA synthesis in newly infected target cells.

### Virion Core Morphology is Affected by NCINIs

**Figure 5:**
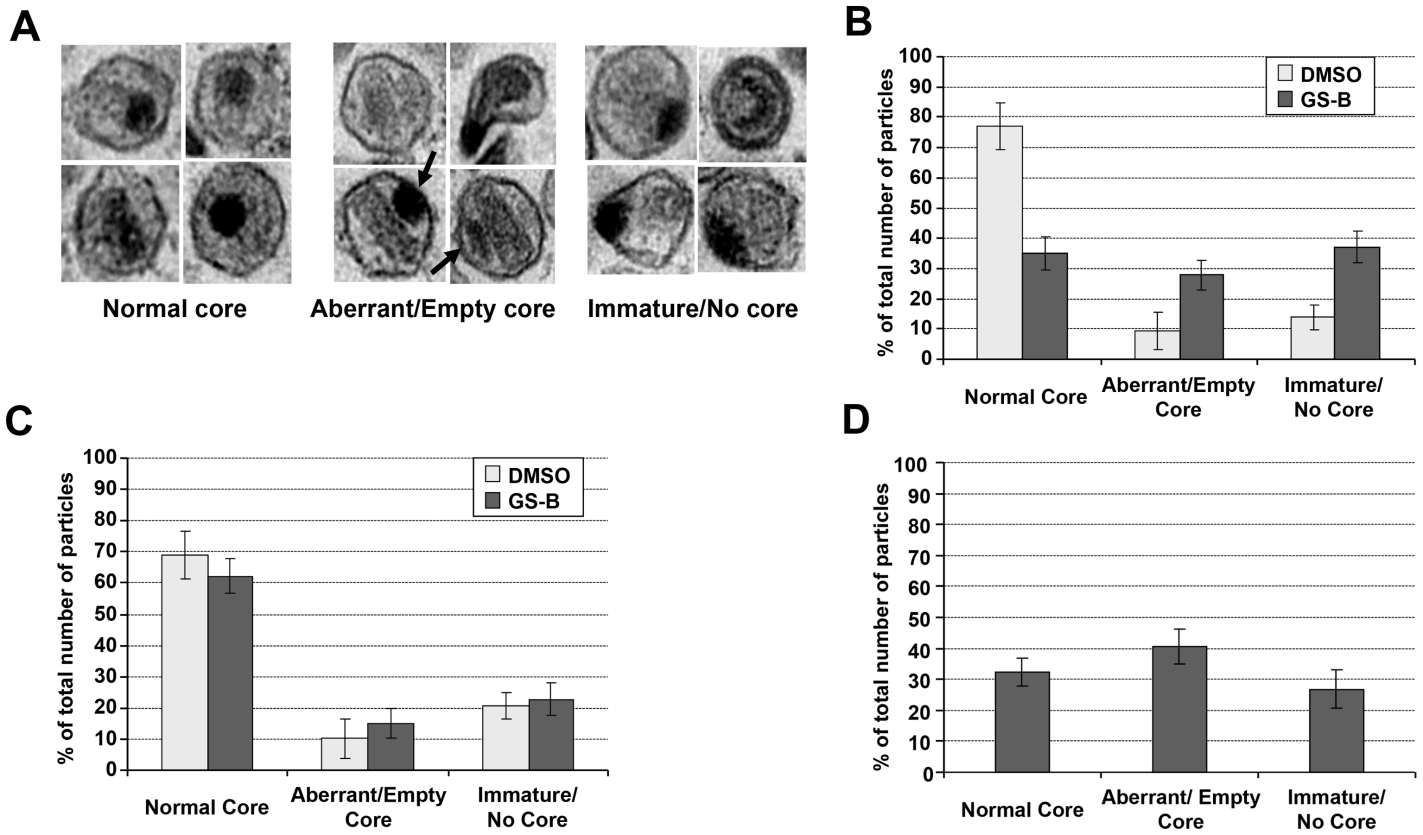
NCINIs disrupt normal HIV-1 core formation and architecture. (**A**) Representative electron micrograph images illustrating normal, aberrant and immature particle morphologies. Arrows highlight the mislocalized electron-dense ribonucleoprotein complex outside of the capsid shell in many aberrant particles. Frequencies of the different core phenotypes are shown for WT (**B**) and T174I mutant (**C**) HIV-1 produced in the presence of DMSO (gray bars) or 1 µM GS-B (black bars). (**D**) Quantitation of core morphology frequencies for the IN-negative virus. For B–D, data represent mean ± SD, each derived from >1000 particles.

We performed thin-section electron microscopy (EM) analysis on purified virus to determine whether NCINIs induced any architectural changes to the particle. Based on the observed core morphology, particles were binned into three phenotypes: i) normal mature conical core, ii) aberrant and/or empty core, and iii) immature and/or no core (Figure 5A). While virus produced in the absence of any inhibitor was composed largely (77%) of particles with normal mature conical cores, this was substantially reduced (to approximately 35%) in virus progeny produced in the presence of 1 µM GS-B (Figure 5B). Importantly, the T174I-resistant virus was minimally affected by treatment with GS-B, and the phenotype distribution mimicked that of mock-treated wild-type (WT) virus (Figure 5C). With WT virus, no novel core phenotypes were observed upon NCINI treatment. Instead, there was a general increase in the typically observed defective particles that either lacked a mature core or had the electron-dense material (likely representing the ribonucleoprotein [RNP] complex) mislocalized outside the empty capsid shell (Figure 5A). Notably, the latter phenotype was previously described for a virus lacking IN [34], an observation that we have independently confirmed. Our quantitative analysis revealed that the Δ-IN virus shows similar frequency of defective core phenotypes as the virus produced in the presence of NCINI (Figure 5D).

### NCINIs Alter HIV-1 Core Yield and Content

**Figure 6:**
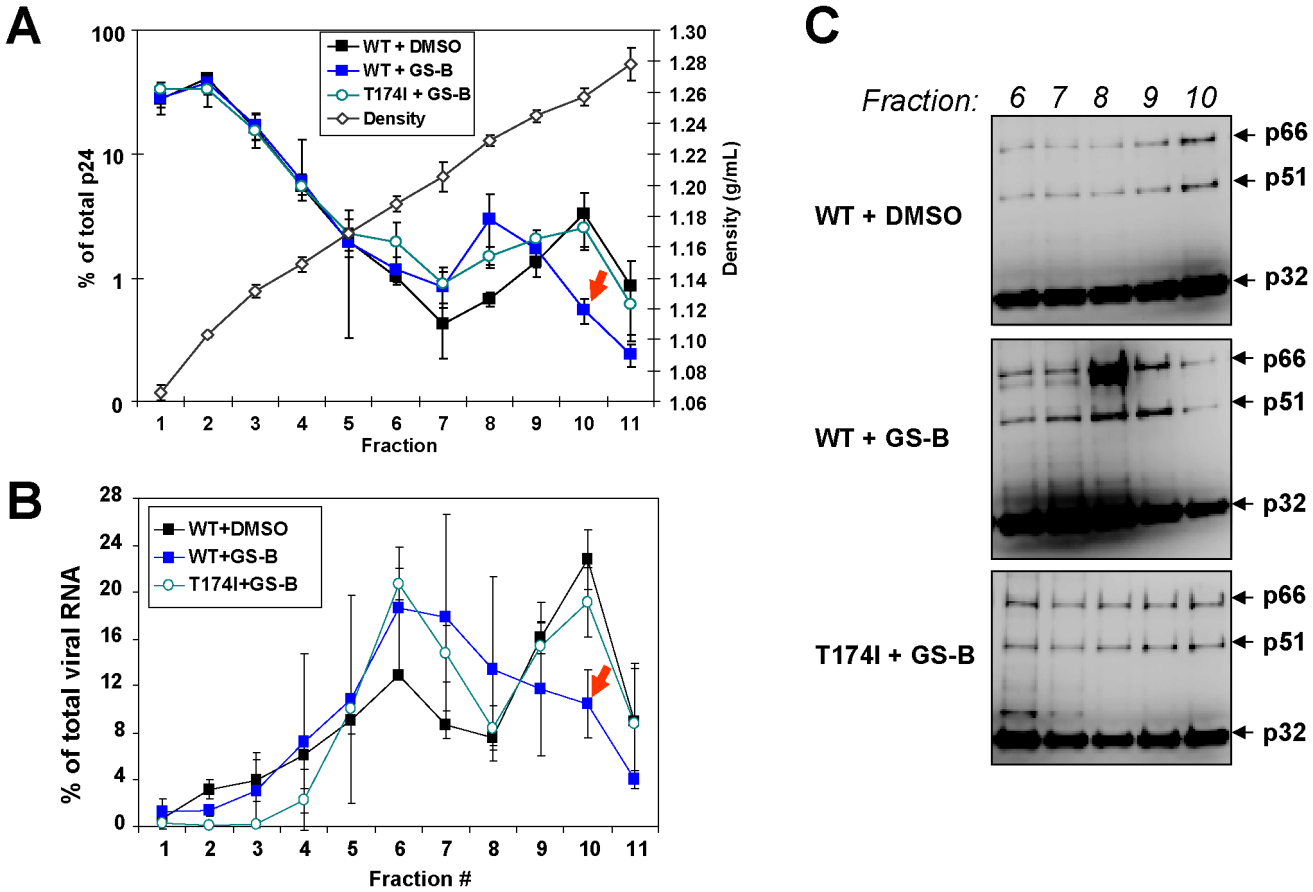
GS-B reduces HIV-1 core yield and content. Wild-type and T174I mutant HIV-1 was produced in the presence of DMSO or GS-B (1 µM) and fractionated over a detergent-layered sucrose gradient. Eleven 3-mL fractions collected sequentially from the top of each gradient were assayed for density (g/mL), CA/IN/RT proteins, and viral nucleic acid content. (A) Distribution of capsid (p24), the major core protein, in relation to sample density. (B) Viral RNA distribution in the gradient fractions, as determined by quantitative RT-PCR. Results in A & B represent the mean ± SD values obtained from four independently fractionated samples. Arrows highlight NCINI-induced reduction of CA and viral RNA within peak core fraction 10. (**C**) Western-blot analyses for relative abundance and distribution of IN (p32) and RT (p51/p66) proteins in gradient fractions 6–10.

To further assess the observed morphological core defects induced by NCINIs, WT and T174I virus produced in the presence and absence of 1 µM GS-B were fractionated over a detergent-sucrose gradient using a previously described ultracentrifugation method [37]. Whereas intact HIV-1 virions sediment to a density of 1.16 to 1.18 g/mL, HIV-1 cores equilibrate at a density of 1.24 to 1.26 g/mL [37]. These densities correspond to fractions 5–6 and 9–10, respectively (Figure 6A). Consistent with particle analysis by EM (Figure 5), p24 content in core fractions 9–10 remained comparable between the WT virus produced in mock-treated cells (4.6% ±1.9% of total p24 content) and T174I virus produced in the presence of GS-B (4.6% ±1.0%), whereas that of WT virus produced in the presence of GS-B was 2-fold lower (2.3% ±0.6%) (Figure 6A). In addition, the WT virus produced in the presence of GS-B had a higher content of p24 in gradient fraction 8. GS-B progeny also exhibited a 2.3-fold reduction of viral RNA within core fractions 9–10 and a corresponding increase in the lower-density fractions (Figure 6B). Western blot profiling showed a similar distribution profile as the viral RNA, with RT and IN proteins shifted towards lighter gradient fractions away from the established core fraction density (Figure 6C). RT distribution determined by enzymatic profiling across the individual fractions matched that observed by Western blot analysis (data not shown).

The contents of fractions 7–8 and 9–10 from each sample were also analyzed by electron microscopy. Consistent with the gradient distribution of p24, conical cores were abundant in samples from the mock-treated WT virus and GS-B-treated T174I mutant virus, but were significantly reduced in abundance, with greater shape variations, in samples from the GS-B-treated WT virus (Figure S1). Fractions 7–8 from each sample showed only very few columnar tubes, with no evidence of conical core-like structures (data not shown). These studies of fractionated progeny particles produced in the presence of NCINIs are consistent with our particle EM analyses and indicate that NCINI treatment disrupts normal core formation and architecture, resulting in an increased prevalence of aberrant cores with a misplaced RNP.

### NCINIs Induce IN Oligomerization within the Viral Particles

**Figure 7:**
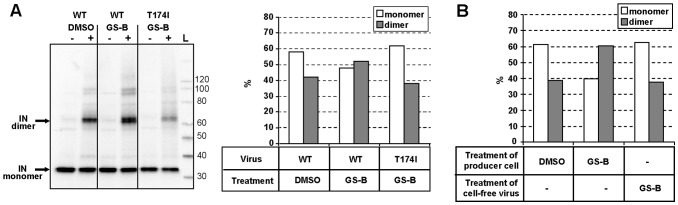
GS-B enhances IN oligomerization during virus production. (**A**) Purified WT or T174I-IN mutant HIV-1 produced in the presence of DMSO or GS-B was briefly treated with cross-linking agent BS3 and the IN monomeric and dimeric forms assessed by anti-IN Western blot analysis. *Left*, Representative anti-IN Western blot of progeny virus treated with cross-linker. *Right*, quantitation of monomeric *vs* dimeric IN forms. (**B**) Quantitation of IN oligomeric state in progeny virus when virus producer cells *vs* cell-free virus particles are treated with GS-B (1 µM). Quantitations represent data from individual representative gels.

Since NCINIs enhance the dimerization of recombinant IN protein [31], [32], [38], we next investigated whether similar changes in the oligomeric state of IN were also induced within virions produced in presence of NCINI. To analyze IN complexes formed, we applied a covalent cross-linking approach previously utilized with purified recombinant HIV-1 IN protein [28] (Figure 7).

The BS3 cross-linker concentration and incubation times were varied to determine optimal assay conditions. Whereas at cross-linker concentrations ≥0.1 mM, a significant proportion of IN was sequestered as large indiscriminate and difficult-to-quantify molecular weight complexes, IN monomer and dimer species were readily detected at 0.04 mM BS3. The analysis of virus produced in the presence of GS-B and cross-linked under optimal conditions revealed a higher proportion of IN dimers compared with virus produced from mock-treated cells (Figure 7A). Importantly, no changes in the IN dimer content were detected in the NCINI-resistant T174I mutant virus produced in the presence of GS-B, a result consistent with the EM and core analysis data. Although the NCINI-induced changes in the monomer-dimer equilibrium were relatively small, the results were reproducible across multiple preparations of virus and multiple distinct NCINIs (data not shown). Interestingly, incubation of mature cell-free virus with GS-B did not induce any changes in the monomer-dimer distribution (Figure 7B), an observation consistent with mature cell-free virions being refractory to NCINI treatment (Figure 1B).

## Discussion

NCINIs have been shown to inhibit integration in target cells [30]. Although this block was thought to occur via inhibition of the IN-LEDGF interactions, more recent data reveal that NCINIs promote and rigidify IN dimers in a manner that impedes the assembly of the IN-vDNA complex [31], [32]. More recently, NCINIs have been reported to also exert antiviral activity during the late stage of the virus replication cycle, although the mechanism involved was not explored [38].

Our work herein reveals important new insights into the NCINI late-stage activity and indicate that their extensively studied antiviral effects on integration in target cells is surprisingly much weaker (up to 100-fold) compared with their impact on virus infectivity following treatment of virus producer cells. We show that NCINI potency is derived primarily during virus production, and not during target cell infection or through virucidal effects on cell-free mature viral particles. Although these compounds act during the same stage of HIV replication cycle as protease inhibitors (PIs), NCINIs are functionally distinct from PIs in that they bind integrase and exhibit no measurable effects on the proteolytic processing of Gag and/or Gag-Pol. Using electron microscopy and biochemical techniques, we further demonstrated that the NCINI-induced defect was manifested at the stage of HIV core maturation and assembly, and correlated with increased IN oligomerization. While these changes do not diminish the ability of these particles to enter new target cells, virus produced in the presence of NCINIs exhibits a potent replication block at the early phase of reverse transcription.

Although a detailed proteomic analysis has not been performed, virus produced in the presence of NCINI appears to be normal for ERT activity and content of genomic RNA as well as major viral proteins. Electron microscopy studies, however, revealed striking defects in both core maturation and architecture, with RNP mislocalization outside of the capsid shell in a significant proportion of virus particles produced from cells treated with NCINIs. These core assembly defects were independently confirmed by gradient ultracentrifugation analysis showing a marked reduction in the frequency of cores with normal density and viral RNA content, along with a shift toward core species with reduced density. Based on the essential role that the HIV core and its proper stability play in the regulation of reverse transcription in target cells [39], these observed core defects in NCINI progeny are likely to contribute significantly to the observed block in vDNA synthesis.

T174I IN was among the resistance mutations selected by GS-A and GS-B and confers high-level resistance to NCINIs [31]. Threonine 174 residue lines the NCINI-binding pocket within the IN dimer interface and makes significant contacts with the inhibitor molecule [31]. Importantly, we show here that an HIV-1 variant with the T174I mutation was resistant to the late-stage inhibitory effect of NCINIs. It is reasonable to conclude that NCINIs exert the late-stage effect in newly produced virions via the same molecular interactions with the IN protein as those involved in mediating the substantially weaker inhibition of vDNA integration in target cells. The significant difference in NCINI potency at the late *vs* early stage is, therefore, intriguing. Possibly, IN interactions with viral and/or host proteins co-packaged within the mature core restrict NCINI access to its binding pocket, either directly or via conformational changes that could reduce the binding affinity of NCNIs for the IN dimer interface. Indeed, a number of host proteins packaged in HIV particles have been shown to bind IN [40], [41]. Alternatively, the early-stage but not the late-stage effect of NCINIs may require their direct competition with LEDGF binding to IN, leading to the potency differential observed herein.

Our observation that neither the overexpression nor the knock-down of LEDGF in the virus producer cells impacted virus infectivity or NCINI antiviral potency implies that the late-stage effect of these compounds does not involve direct competition with LEDGF binding to the IN domain during virus assembly. Although its essential role in vDNA integration has been firmly established, there is little evidence for the requirement of LEDGF during the production of infectious virus. A substantial (>90%) knockdown of LEDGF in virus-producing cells was reported to minimally impact virus production or its infectivity [12], and proteomic studies did not identify LEDGF among host proteins packaged within HIV-1 particles [40], [41]. In addition, the progeny virus harboring a Q168A mutation in IN that disrupts its binding to LEDGF was replication defective due to a block in vDNA integration, but the mutant virions showed no late-stage defects and supported normal reverse transcription in target cells [42]. Furthermore, although cell lines overexpressing the dominant negative LEDGF-derived IN binding domain (IBD) were refractory to viral infection due to failed integration, progeny virus produced from the IBD overexpressing cells was reported to be fully infectious [43]. Combined, these observations indicate that LEDGF does not serve any essential role in the late stage of the HIV life cycle. The NCINI-induced effects on HIV core assembly and particle infectivity are, therefore, unlikely to be related to the ability of the compounds to inhibit LEDGF-IN interaction, supporting a LEDGF-independent inhibitory mechanism of these antivirals.

Detailed biochemical studies from independent groups have converged on the finding that NCINIs promote IN multimerization, locking the IN subunits in a complex with a more rigid conformation [31], [32]. This promotion of nonproductive multimerization was proposed to constitute the underlying mechanism behind the compound-induced block of integration in target cells, namely by preventing the binding of IN to vDNA and/or blocking its interaction with LEDGF. This is supported by recent findings by Feng et al. [44] that the NCINI resistance-conferring A128T mutation perturbs the ability of NCINIs to bind and promote IN multimerization with minimal impact on IN-LEDGF binding. We used a chemical cross-linking approach to demonstrate that IN oligomerization is also modified in virions produced in the presence of NCINIs. This could make IN unavailable for facilitating core assembly and/or maturation. Interestingly, similar core morphology defects were exhibited by viral particles produced in the presence of NCINIs and those lacking the whole IN domain. NCINIs were ineffective at promoting IN dimerization in fully mature cell-free virus, again suggesting that within the mature particle, IN remains either conformationally inaccessible or physically protected via interactions with other components of the RNP complex.

The previously described IN-negative as well as IN class II HIV mutants have been shown to be defective in reverse transcription in the target cells, although no effects on particle production or Gag-Pol processing were observed [45]. Consistently, we have shown that both the IN-negative and the class II IN mutant viruses (L241A or K258A) exhibited a clear block at the early stages of reverse transcription, a phenotype shared by viruses produced in the presence of NCINIs (Figure S2). IN protein, however, appears to be dispensable for virion-associated reverse transcriptase activity as mature cell-free virions lacking IN contain enzymatically functional RT complex [46]. Interestingly, our data also demonstrate normal enzymatic activity of endogenous RT in the otherwise noninfectious virions produced in the presence of NCINIs. In the case of the HIV variant with IN mutation C130S that exhibits a block at the reverse transcription step, biochemical findings suggest a disruption of the direct IN–RT interaction as a potential cause [47]. HIV-1 IN and RT have been shown to physically interact *in vitro*, and an RT-binding domain has been mapped to the IN C-terminal domain [47], [48]. However, a role for this interaction has not been confirmed in the context of intact mature virions or in the target cell. Together, these observations indicate that IN protein does not directly affect the virion-associated enzymatic activity of RT, but do not exclude the direct interaction between IN and RT proteins and its potential role in the proper assembly and/or encapsidation of the RNP complex within the maturing virions.

In summary, our studies have revealed an unexpected and novel mechanism by which NCINIs block the replication of HIV. At the time of completion of our report, Jurado *et al*. [33] published a study focused on the mechanistic characterization of allosteric HIV integrase inhibitors analogous to NCINIs. This independent study arrived at virtually identical conclusions, demonstrating a potent late-stage antiviral effect of ALLINIs due to their interference with proper virus assembly, leading to a block of reverse transcriptase in newly infected target cells. Similar to us, Jurado *et al*. [33] linked this mechanism to the ability of ALLINIs to induce oligomerization of IN within HIV particles undergoing the maturation process. This remarkable consistency of both reports solidifies the current understanding of the antiviral effect of NCINIs/ALLINIs and underscores the continuing interest in further optimization and subsequent clinical development of these compounds as a novel class of antiretrovirals with a unique orthogonal mechanism of action.

## Materials and Methods

### Materials

GS-A, GS-B, and RAL were synthesized as previously described [31]. HIV-1 inhibitors used were as follows: efavirenz (EFV, A603765), nevirapine (NVP, A618440) and atazanavir (ATV, A790051) from Toronto Research Chemicals (Toronto, Canada), entry inhibitor AMD3100 (Catalog No. A5602) and virucidal agent PD-404,182 (Catalog No. P2742) from Sigma (St. Louis, MO) and fusion inhibitor C34 (Catalog No. 62101) from AnaSpec (Freemont, CA). Purified HIV-IIIB virus and anti-CA antibody were purchased from Advanced Biotechnologies (Columbia, MD). Anti-IN and -RT antibodies were purchased from Abcam (Cambridge, MA) and MyBioSource (San Diego, CA), respectively.

### Vectors and Cloning

Plasmid pKS13 is a NL4.3-based vector in which *vpr* and *env* expression were knocked out by inserting a ‘T’ at the AflII site and two bases (TA) at the NdeI site, respectively, causing frame shifts in both open reading frames. A codon-optimized firefly luciferase gene was introduced in place of the *nef* coding sequence by replacing the BamHI-NcoI fragment. KS13-IN-T174I, KS13-IN-L241A, and KS13-IN-K258A carrying point mutations in the IN coding region were generated by site-directed mutagenesis (Catalog No. 200521, Stratagene, Santa Clara, CA). The KS13-ΔIN was generated as previously described [34]. The pLet-Lai vector encoding the CXCR4 envelope was kindly provided by Vicente Planelles (University of Utah, Salt Lake City) [49]. Plasmid pHCMV-G encoding the vesicular stomatitis virus G (VSV-G) envelope protein has been previously described [50]. To generate the β-lactamase-Vpr construct, the β-lactamase gene fused to the N-terminus of Vpr was synthesized (DNA2.0, Menlo Park, CA) and inserted into the pcDNA3.1(+) mammalian expression vector (Invitrogen, Grand Island, NY) via BamHI/XhoI ligation, as previously described [36].

### Antiviral Assays

The 5-day cytoprotection antiviral assay in MT-2 cells infected with HIV-1 IIIB has been previously described [51]. Single-cycle assays were set up such that compound was present selectively at the early stage, late stage, or during the full course of infection. Briefly, HEK293T cells were co-transfected with the KS13 vector along with envelope vector pHCMV-G in T-75 flasks. Sixteen hours post-transfection, cells were replated in 96-well plates either in the absence or presence of serial dilutions of compound. Cell-free culture supernatants were harvested 48 hours post-transfection and diluted 10,000-fold with media. Fifty microliters was added to 50,000 MT-2 cells in 50 µL of media in the absence or presence of serially diluted compound. All infections were performed in triplicate wells of a 96-well plate. Three days post-infection, luciferase signal was developed using One-Glo reagent (Promega, Madison, WI) per the manufacturer’s instructions, and plates were read using an Envision plate reader (Perkin-Elmer, Waltham, MA). Data analysis was performed using GraphPad Prism to calculate EC_50_s.

### Virucidal Assays

KS-13 virus was mixed with DMSO or a small molecule inhibitor in complete DMEM medium and incubated for 2 hours at 37°C. Virus samples were then repeatedly diluted and concentrated three times over a Vivaspin 20 centrifugal concentrator (100 k MWCO, Sartorius, Bohemia, NY) to remove free compound. The procedure resulted in an estimated 8×10^6^ effective dilution of the compound. The purified virus samples were brought up to their original volume using complete DMEM and used to infect MT-2 cells. Infections were performed in triplicate wells of a 96-well plate with 3×10^4^ cells per well. Approximately 48 hours post-infection, luciferase signal was measured as described earlier.

### LEDGF Overexpression and Knock-down in Virus Producer Cells

LEDGF overexpression was achieved through transient transfection of HEK293T cells with the pCMV6-XL5-LEDGF (Catalog No. SC119664, Origene, Rockville, MD). HEK293T cells were co-transfected with the virus plasmids KS-13 and pHCMV-G along with pCMV6-XL5-LEDGF or the empty vector pcDNA3.1 (2∶1:1 ratio respectively) using Lipofectamine 2000 per the manufacturer’s protocol. Cells were harvested 12, 24, 36, and 48 hours post-transfection, and LEDGF expression levels were monitored by Western blot analyses and quantified using IQ software (GE Healthcare). To measure compound EC_50_s, co-transfections were performed in T75 flasks. Sixteen hours post-transfection, cells were trypsinized, washed, and replated in 24-well plates in the presence of NCINI or control compounds. Twenty-four hours post-transfection, cells were refed with fresh compound-containing media. Virus-containing supernatant was harvested another 24 hours later. Harvested supernatants were diluted 5,000-fold and used to infect MT-2 cells as described earlier.

LEDGF knockdown in HEK293T cells was achieved by stable transfection using LEDGF-targeting lentiviral shRNA constructs from Open Biosystems (Catalog No. RHS4531-EG11168, Thermo Scientific, Rockford, IL) and the Trans-Lentiviral shRNA packaging kit (Catalog No. TLP5912, Thermo Scientific, Rockford, IL) per the manufacturer’s protocols. Vector with scrambled siRNA sequence was used as a control. Virus supernatants harvested 48 hours post-transfection were clarified and used to transduce HEK293T cells in 6-well plates. Puromycin (5 µg/mL) selection was initiated 24 hours post-infection, and stably transfected cells were expanded under selection. LEDGF expression levels in the established cell lines were assessed by Western blot. For virus production, the established cell lines were co-transfected with pKS13 and pHCMV-G plasmids. Compound EC_50_s were measured using an identical approach as described for the LEDGF overexpression system.

### Endogenous Reverse Transcriptase (ERT) Assay

Virus produced in the presence of compounds or DMSO was concentrated over a 20% sucrose cushion, and ERT activity assayed as previously described [52], with slight modifications. Reactions were performed in 50 µL final volume and contained 50 mM Tris (pH 7.8), 60 mM KCl, 5 mM MgCl_2_, 10 mM NaCl, 10 mM DTT, 0.5 mM EDTA, 0.005% Nonidet P-40, 0.4 mM of each dATP, dGTP, and dTTP, 10 mM dCTP, 10 mCi of [α-^33^P]dATP, and equal amounts of virus, based on p24. Reactions were incubated at 37°C. At required time points, duplicate 10 µL samples were blotted onto a DEAE filter paper (Wallac), dried, and processed as previously described [53].

### Virus Entry Assay

HEK293T cells were co-transfected with KS13, pLet-Lai, and pBlaM-Vpr in the presence and absence of 1 µM GS-B. Clarified virus supernatants were prepared 48 hours later and concentrated using PEG-IT (Catalog No. LV825A-1, System Biosciences, Mountain View, CA). The virion-based fusion assay was performed essentially as described previously [36]. Briefly, MT-2 cells (1×10^5^) were infected with 400 ng p24 equivalents of virus produced in the presence of DMSO, 1 µM AMD3100, and 1 µM GS-B or 1 µM EFV and incubated for 2 hours at 37°C. Cells were then washed in CO_2_-independent medium (Gibco, Rockville, MD) and loaded with CCF2/AM dye for 1 hour at room temperature per the manufacturer’s protocol (Catalog No. K1095, Invitrogen). Cells were washed twice and resuspended in 200 µL of CO_2_-independent media containing 10% FBS and 2.5 mM probenecid (Sigma), and the β-lactamase reaction was allowed to develop for 16 hours at room temperature in the dark. Cells were washed in PBS then fixed in a 1.2% solution of paraformaldehyde for 4 hours at 4°C. The change in emission fluorescence of CCF2 dye after cleavage by BlaM-Vpr was measured using an LSRFortessa flow cytometer (Becton Dickinson, Franklin Lakes, NJ). Data were analyzed using FlowJo software (TreeStar, Ashland, OR).

### Quantification of HIV DNA

HEK293T cells were co-transfected with pKS13 and pHCMV-G plasmids in the presence of DMSO, 1 µM GS-B, and 1 µM RAL or 1 µM EFV. Seventy-two hours post-transfection, clarified virus supernatants were treated with DNase (30 U/mL) in the presence of 5 mM MgCl for 90 minutes at 37°C to eliminate any plasmid DNA in the samples. Equal volumes (1.5 mL) of virus supernatants were used to infect MT2 cells in duplicate wells (2×10^6^ per well) by spinoculation (1,200 ×g, 2 hours) in a total volume of 2 mL DMEM supplemented with 6 µg/mL polybrene. To synchronize the infection, cells were washed extensively to remove unbound free virus and resuspended in fresh media with compound. Cell culture plates were then transferred to a humidified 37°C incubator. To account for signal from residual contaminating plasmid, a few DNase-treated DMSO virus samples were heat-inactivated for 1 hour at 65°C, and infections were set up in parallel to facilitate background correction during data analysis. Cells were harvested at 12 hours for early and late RT product quantification and at 24 hours for 2-LTR and Alu-LTR product quantification. Viral DNA was isolated using a QIAamp DNA mini kit (Qiagen, Catalog No. 51304) and quantified using TaqMan real-time PCR using the ABI Prism 7900HT sequence detection system (Applied Biosystems, Foster City, CA). Primer-probe sets used were as follows: Early RT products, RU5-F (5′-TCTGGCTAACTAGGGAACCCA-3′), RU5-R (5′-CTGACTAAAAGGGTCTGAGG-3′), and RU5 probe (5′-6FAM-TTAAGCCTCAATAAAGCTTGCCTTGAGTGC-6TAMSp-3′); Late RT products, PBS-F (5′-TTTTAGTCAGTG TGGAAAATCTCTAGC-3′), PBS-R (5′-TTGGCGTACTCACCAGTCGCC-3′), and PBS probe (5′-6FAM-TCGACGCAGGACTCGGCTTGCT-6TAMSp-3′). Primer-probe sets for 2-LTR circles, Alu-LTR integration junctions, and the host β-globin gene (used to normalize for cell number) were as previously described [54].

### HIV-1 Fractionation and Core Analyses

WT and IN-T174I mutant KS13/X4 viruses produced in the presence and absence of 1 µM GS-B were concentrated 40-fold. To analyze virus cores, concentrated virus samples were fractionated over a detergent-layered sucrose gradient as previously described [37] with minor modifications. Equilibrium gradients were prepared by sequential step-wise overlays of 6 mL each of 70%, 60%, 50%, 40%, and 30% sucrose in STE buffer (10 mM Tris-HCl [pH 7.4], 100 mM NaCl, 1 mM EDTA) in ultra-clear Beckman ultracentrifugation tubes. Gradients were sequentially overlaid with 0.75 mL cold 15% sucrose containing 0.25% TritonX-100, 1.5 mL cold 7.5% sucrose-STE, and 1.5 mL concentrated virus (∼20 µg total p24). Samples were fractionated in an SW28 rotor at 28,000 rpm for 23 hours at 4°C, after which eleven 3 mL fractions were sequentially collected from the top of each tube. The density of each fraction was determined using a refractometer (Catalog No. 13950000, Reichert, Depew, NY), and p24 content was quantified by ELISA (Catalog No. NEK050B001KT, Perkin-ElmerViral RNA was isolated from 160 µL aliquots of each fraction (Catalog No. 52904, Qiagen) and quantified by qRT-PCR (TaqMan RNA-to-CT 1-Step Kit, Catalog No. 4392938, Applied Biosystems) using late-RT primers and probe as described above. Quantities of contaminating DNA were determined in the absence of the RT step and subtracted from all qRT-PCR-determined measurements. The RT activity in each fraction was quantified using an ELISA-based RT colorimetric assay (Catalog No. 11468120910, Roche Diagnostics, Indianapolis, IN). RT and IN content in each fraction were assessed by Western blot analyses after protein was pelleted from 1.2 mL of each fraction by ultracentrifugation (Beckman Ti50.4 rotor, 32,000 rpm, 2 hours at 4°C). Resuspended pellets were subjected to SDS-PAGE and Western blot analysis using mAbs to HIV-1, integrase, and RT.

### IN Cross-linking Assays

Purified virus (50 ng of p24 equivalents) in PBS/0.25% Triton X-100 was treated with 0.05 mM bis(sulfosuccinimidyl)suberate (BS3) cross-linking agent (Catalog No. 21580, Thermo Scientific, Rockford, IL) in a final volume of 12 µL for 20 minutes at room temperature. Reactions were quenched by addition of Tris-HCl pH 7.5 to a final concentration of 50 mM and incubating for an additional 20 minutes. Complexes were resolved by reducing SDS-PAGE (4–12% Bis-Tris Gel; Invitrogen), probed by Western blot using anti-IN mAb, and quantified using IQ software.

### Electron Microscopy Analysis

Virus pellets were prepared in 1.5 mL microfuge tubes by overlaying purified concentrated virus on 20% sucrose and spinning at 14,000 rpm for 2 hours at 4°C. Supernatant was removed, and viral pellets were fixed in 2% glutaraldehyde and 1% paraformaldehyde in 0.1 M sodium cacodylate buffer pH 7.4, post-fixed in 2% osmium tetroxide in the same buffer, en bloc stained in 2% aqueous uranyl acetate, dehydrated in acetone, infiltrated, and embedded in LX-112 resin (Ladd Research Industries, Burlington, VT). Samples were ultrathin sectioned and counter stained with 0.8% lead citrate. Grids were examined on a JEOL JEM-1230 transmission electron microscope (JEOL USA, Inc., Peabody, MA) and photographed with the Gatan Ultrascan 1000 digital camera (Gatan, Inc., Warrendale, PA). For EM analysis of purified cores, 0.6 mL from each of fractions 7–8 and 9–10 were diluted with 0.6 mL cold STE and concentrated at 32,000 rpm for 2 hours at 4°C in a Beckman Ti50.4 rotor. Pelleted material was resuspended in 50 µL PBS and fixed in 1% glutaraldehyde. Samples were stored at 4°C until immobilized on carbon-coated grids and processed for negative staining EM as previously described [55].

## Supporting Information

Figure S1 **NCINIs alter HIV core abundance.** Representative morphologies of purified HIV-1 core-like structures (Fig. 6, fractions 9 and 10) from different producer cell treatments are shown. *Left*, WT+DMSO; *middle*, WT +1 µM GS-B; *right*, T174I-IN +1 µM GS-B. The number of core-like structures (mean ± SD) observed per micrograph over at least six micrographs photographed at 8,000× magnification were 6.0±2.4 (WT+DMSO), 0.5±0.5 (WT +1 µM GS-B) and 5.5±7.0 (T174I-IN +1 µM GS-B).
(TIF)

Figure S2 **IN-negative and Class II integrase mutants are defective in vDNA synthesis.** Quantitative PCR assessment of early-RT (grey bars) and late-RT (white bars) products in MT-2 target cells directly infected with IN-negative, L241A-IN, or K258A-IN mutant virus. Results represent mean ± SD values normalized to WT virus (set to 100%) obtained from quadruplicate infections each assayed in duplicate.
(TIF)
