# Supplementary figures and images for: Non-Catalytic Site HIV-1 Integrase Inhibitors Disrupt Core Maturation and Induce a Reverse Transcription Block in Target Cells
Source: PLoS One. 2013 Sep 9;8(9):e74163. doi: 10.1371/journal.pone.0074163 (PMC3767657; doi:10.1371/journal.pone.0074163)

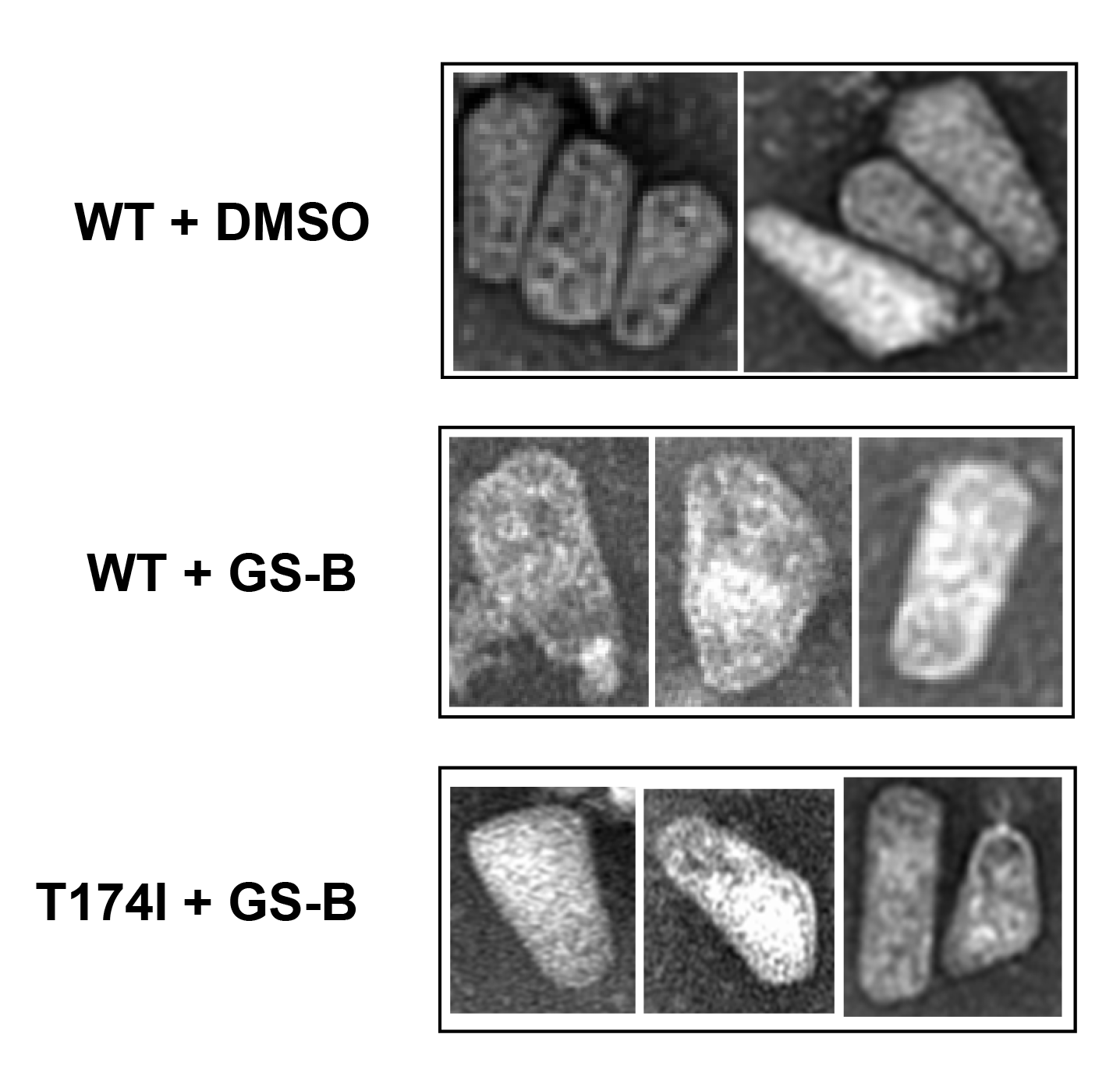

Supplement: Figure S1 — NCINIs alter HIV core abundance. Representative morphologies of purified HIV-1 core-like structures (Fig. 6, fractions 9 and 10) from different producer cell treatments are shown. Left, WT+DMSO; middle, WT +1 µM GS-B; right, T174I-IN +1 µM GS-B. The number of core-like structures (mean ± SD) observed per micrograph over at least six micrographs photographed at 8,000× magnification were 6.0±2.4 (WT+DMSO), 0.5±0.5 (WT +1 µM GS-B) and 5.5±7.0 (T174I-IN +1 µM GS-B). (TIF) [file pone.0074163.s001.tif]

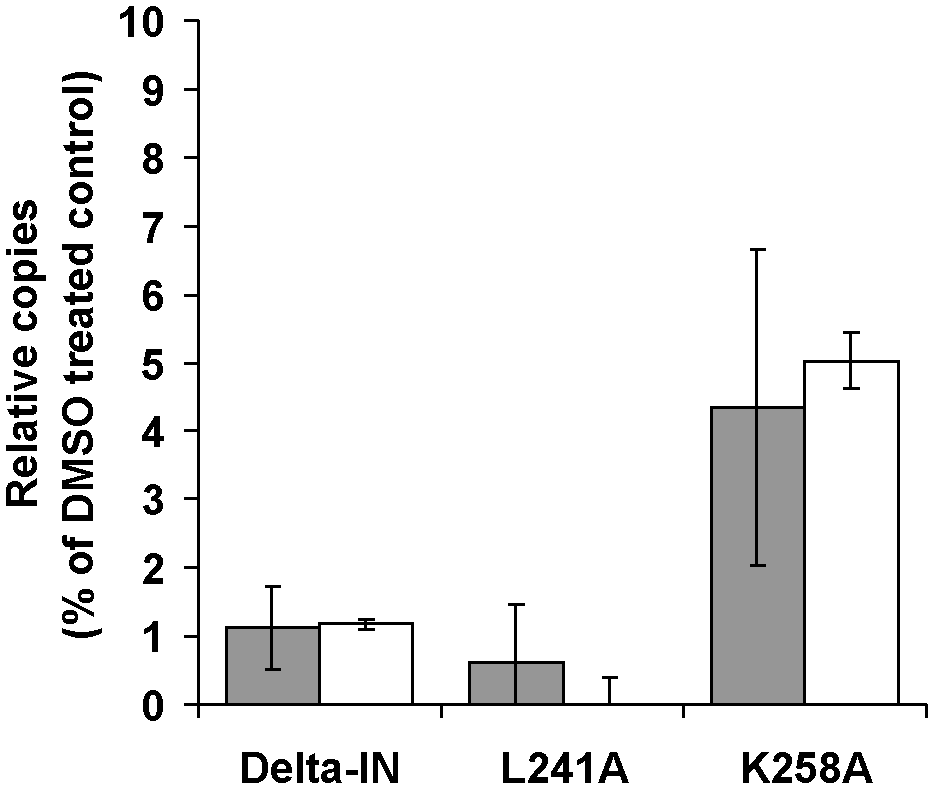

Supplement: Figure S2 — IN-negative and Class II integrase mutants are defective in vDNA synthesis. Quantitative PCR assessment of early-RT (grey bars) and late-RT (white bars) products in MT-2 target cells directly infected with IN-negative, L241A-IN, or K258A-IN mutant virus. Results represent mean ± SD values normalized to WT virus (set to 100%) obtained from quadruplicate infections each assayed in duplicate. (TIF) [file pone.0074163.s002.tif]
